# Interaction between a variant of chromosome 9p21.3 locus and diet antioxidant capacity on metabolic syndrome in Tehrani adults

**DOI:** 10.1186/s13098-018-0372-z

**Published:** 2018-10-18

**Authors:** Atieh Mirzababaei, Mehdi Mollahosseini, Mohammad Hossein Rahimi, Mir Saeed Yekaninejad, Zhila Maghbooli, Reza Sobhani, Khadijeh Mirzaei

**Affiliations:** 1 Department of Community Nutrition, School of Nutritional Sciences and Dietetics, Tehran University of Medical Sciences (TUMS), P.O. Box: 14155-6117, Tehran, Iran; 2 Department of Epidemiology and Biostatistics, School of Public Health, Tehran University of Medical Sciences (TUMS), Tehran, Iran; 3 Endocrinology and Metabolism Clinical Sciences Institute, Tehran University of Medical Sciences (TUMS), Tehran, Iran

**Keywords:** CDKN2B, Oxygen radical absorbance capacity, Antioxidants, Metabolic syndrome, Cardio-vascular diseases, Nutrigenomics

## Abstract

**Background:**

Genome-wide association studies have shown that risk alleles on chromosome 9p21.3 locus, are associated with increasing the risk of cardiovascular diseases (CVDs). Several epidemiological studies have found that metabolic syndrome (MetS) is associated with CVDs. Dietary antioxidants also have shown to have potential favorable effects on MetS prevention. This study examined the interactions between rs1333048 genotypes on 9p21 genetic region and Total antioxidant capacity (TAC) on odds of MetS.

**Methods:**

263 Tehrani adults were enrolled in this cross-sectional study. The MetS was defined according to the ATPIII. Dietary intake was assessed daily using a FFQ with 147 items. Dietary TAC was assessed according to United States Department of Agriculture database for oxygen radical absorbance capacity (ORAC). Bioelectrical impedance analysis method was used for body analysis and rs1333048 were genotyped by restriction fragment length polymorphism method. Participants were categorized into three groups based on rs1333048 genotypes.

**Results:**

The results demonstrate that, prevalence of C allele was 52.85% and A allele was 47.15%. After adjustment for confunder variable, this study demonstrated an interaction between AA genotype and high Lyophilic oxygen radical absorbance capacity (L-ORAC) and high Hydrophilic oxygen radical absorbance capacity (H-ORAC) intake on low odds of MetS (OR = 0.24, 95% CI = 0.06–0.94, P for interaction = 0.04, OR = 0.26, 95% CI = 0.06–0.99, P for interaction = 0.04). Also, our result indicated, there was no interaction between AA genotype and high total oxygen radical absorbance capacity (T-ORAC) and total phenolic intakes on reduce odds of MetS (OR = 0.07, 95% CI = 0.07–1.10, P for interaction = 0.07, OR = 0.58, 95% CI = 0.16–2.07, P for interaction = 0.40) respectively.

**Conclusion:**

The results of the present study indicate that high L-ORAC and high H-ORAC intake may modify the elevated odds of MetS in AA genotype of rs1333048 on the 9p21 genetic locus.

## Background

Metabolic syndrome (MetS) is a growing public health problem worldwide, one which increases the risk of the development and progression of cardiovascular diseases (CVDs) [1]. MetS and its components, including hyperglycemia, hypertension, dyslipidemia and abdominal obesity have been indicated to be strongly associated with oxidative stress; a condition which is characterized by excessive production of reactive oxygen species (ROS) and/or lipid peroxidation, as well as diminished antioxidant protection. Previous studies have shown increased production of oxidant biomarkers and decreased levels of antioxidant defense in patients with MetS. Oxidative stress impairment or altered antioxidant status have been suggested as effective keys in the onset of certain chronic diseases such as CVDs, MetS [2]. Moreover, oxidative stress seems to be associated with the development of chronic heart diseases (CHDs) and metabolic complications among patients with MetS [3]. Increased oxidative stress underlies the pathophysiology of hypertension [4] and CVDs [5] by directly affecting vascular wall cells and decreases insulin secretion from pancreatic β cells [6].

Reports indicate that diet antioxidants can protect against oxidative damage and related inflammatory complications [7]. Thus, a high intake of antioxidants is associated with reduced mortality [8]. Since that the concentration of single antioxidants may not reflect the total antioxidant power of food, as well as other possible interactions or synergetic effects of antioxidants, the concept of total antioxidant capacity (TAC) was introduced for investigating the beneficial effects of dietary antioxidants occurring in mixed diets as well as an approach to food characterization [9–11]. The dietary TAC describes the ability of food antioxidants to scavenge free radicals, and it is measured using the oxygen radical absorbance capacity (ORAC) assay. Recently cross-sectional studies reported that dietary TAC positively associated with plasma total antioxidant capacity [12, 13].

In addition to environmental factors (including lifestyle) that can increase the risk of CVDs, it can also be influenced by genetic variants and several genomic regions have been linked to risk of CVDs [14]. Genome-wide association studies (GWAS) have been successful in identifying the same susceptibility locus on chromosome9p21 that associate with multifactorial diseases. Studies indicate that the 9p21 alleles contributed to CVDs risk by stimulating ectopic fat accumulation, hypertension and impaired glucose metabolism [15, 16]. Some of these studies have investigated the possible association between single nucleotide polymorphisms (SNPs) at this locus with MetS and its components as important risk factors for CVDs as well as their interaction with environmental factors such as dietary intake [17]. Overall, diet plays an important role in the development of CHDs, several studies showed that low consumption of fruits, vegetables and fish which contains high antioxidants and protects the heart are associated with CVDs [18] and changes in diet clearly have modified CVDs risk factors [19]. Today, knowledge of how such diet related risk factors may interact with genetic susceptibility variants on CVDs risk is important for CVDs prevention.

However, based on our search no available study has evaluated the interaction between these SNPs and dietary antioxidant capacity on MetS and its components. In this study, the interactions between dietary TAC and chromosome 9p21 polymorphism rs1333048 on odds of MetS were investigated.

## Methods and materials

### Study population

In this cross-sectional study, 363 participants (18–55 years) were enrolled. Sampling was done with advertising in the city. Individuals were included if they met following criteria: namely age 18–55, no smoking and alcohol. Participants with a history of CVDs, diabetes, cancer or stroke were excluded because of possible disease-related changes in diet and who were taking any therapeutic medications. We also excluded subjects whose reported daily energy intakes were 800 kcal/day (3347 kJ/day) or 4200 kcal/day (17 573 kJ/day) [27]. These exclusions left 263 subjects (125 men and 138 women) for the current analysis. Each participant was completely informed about the study protocol and provided a written and informed consent form before taking part in the study. The study protocol has approved by the ethics committee of Endocrinology and Metabolism Research Center of Tehran University of Medical Sciences (TUMS) with the following identification: 93-04-161-27-722-1495-80.

### Measurement of biochemical parameters

All blood samples were collected at 8:00 to 10:00 A.M. after having 8–12 h fasting state at the EMRC laboratory of Shariatei hospital of TUMS. To collect serums, Serum samples were centrifuged 10 min at 3000 rpm, aliquoted into 1 ml tubes and stored at − 80 until they were analyzed. Fasting plasma glucose (FPG) was measured on the day of blood collection by glucose oxidase phenol 4-aminoantipyrine peroxidase (GOD/PAP) method. Serum triglycerides (TG) concentrations were assayed with triacylglycerol kits (Pars Azmoon Inc, Tehran, Iran) by using glycerol-3-phosphate oxidase phenol 4-aminoantipyrine peroxidase (GPOPAP) method. Total cholesterol levels were measured by the Enzymatic Endpoint method and direct high- and low-density lipoprotein was measured by enzymatic clearance assay. Serum hyper sensitive C-reactive protein (hsCRP), as a sensitive marker of inflammation, was measured by an immunoturbidimetric assay (Randox laboratories kit, Hitachi LTD, Tokyo, Japan). Serum insulin concentrations were analyzed through enzyme-linked immunosorbent assay (ELISA) method (Human insulin ELISA kit, DRG Pharmaceuticals, GmbH, Germany), and the minimum detectable concentration was 1.76 U/ml, Intra CV was 2.19% and Inter CV was 4.4%.

### Anthropometric measurements

Weight was measured while the subjects were minimally clothed and not wearing shoes. Weight was measured to the nearest 100 g by using digital scales. Height was measured by using a tape measure while the subject was in a standing position and not wearing shoes, and the shoulders were relaxed. Body mass index (BMI) was also calculated using the “weight (kg)/height^2^ (m^2^) “equation. Blood pressure (BP) was measured using a standardized sphygmomanometer after 5 min of rest. Waist circumference (WC) was measured in the middle point of iliac crest and rib cage.

### Complete body composition analysis

Participant’s body composition was assessed through Body Composition Analyzer BC-418MA-Tanita (United Kingdom). This Bioelectrical Impedance Analyzer (BIA) is designed send out a very weak electric current to measure the impedance (electrical resistance) of the body. We followed all of the following instructions for an accurate measurement. To prevent a possible discrepancy in measured values, before assessing body composition, Participants were asked not to exercise vigorously, carry out any electric device and intake excessive fluid or food; they were performed in the morning in a fasting condition and urinate just before body composition analysis to get a more accurate result of the measurements.

### Dietary assessment and dietary TAC calculation

Dietary data was collected using a validated semi-quantitative food frequency questionnaire (FFQ) with 147 food items. Trained dietitians asked participants to designate their intake frequency for each food item consumed during the past year on a daily, weekly or monthly basis. Portion sizes of consumed foods were reported in household measures, and were then converted to grams [20].

Due to the fact that the Iranian Food composition table (FCT) is incomplete, and has limited data on the nutrient content of raw foods and beverages, the US Department of Agriculture (USDA) FCT [21] was used in order to analyze foods and beverages for their energy and nutrient content. Dietary TAC describes the ability of food antioxidants to scavenge free radicals, and it is measured using ORAC assay [22]. The ORAC is one of the common methods to evaluate the antioxidant capacity [38]. The antioxidant capacity of lipophilic and hydrophilic antioxidants in the samples are evaluated using Lyophilic-oxygen radical absorbance capacity (L-ORAC) and Hydrophilic-oxygen radical absorbance capacity (H-ORAC) methods, respectively, and their total is used as an indicator of the dietary antioxidant capacity of food. Antioxidants can be physically classified by their solubility into two groups: (a) lipophilic antioxidants such as vitamin E and carotenoids and (b) hydrophilic antioxidants such as vitamin C and the majority of polyphenolic compounds [39]. The ORAC values were utilized to develop individual indices for TAC in the following manner: H-ORAC, LORAC, total-oxygen radical absorbance capacity (T-ORAC) and total phenolic (TP). H-ORAC, L-ORAC, and T-ORAC are reported in μmol of Trolox equivalents per 100 grams (μmolTE/100 g), while TP is reported in mg gallic acid equivalents per 100 g (mgGAE/100 g) [23].

### The HOMA-IR calculation

Homeostatic model assessment-insulin resistance (HOMA-IR), was calculated according to the following equation: [fasting plasma glucose (mmol/l) × fasting plasma insulin (mIU/l)]/22.5 [24].

### Definition of metabolic syndrome and its components

Cardio-metabolic risk factors for metabolic syndrome were defined according to the diagnostic criteria proposed by Adult Treatment Panel III (ATP III) [25] and new cutoff points of WC for Iranian adults [26]; the syndrome was characterized as having at least 3 of the following metabolic abnormalities: (1) Hyperglycemia as FPG ≥ 100 mg/dl (5.6 mmol/l); (2) Hypertriglyceridemia as serum TG ≥ 150 mg/dl (1.69 mmol/l); (3) Low HDL-C serum < 40 mg/dl (1.04 mmol/l) for men, and < 50 mg/dl (1.29 mmol/l) for women; (4) Hypertension as BP ≥ 130/85 mmHg; and (5) Abdominal adiposity (defined as waist circumference 88 cm [women] or 102 cm [men]).

### DNA extraction and gene sequencing

Single nucleotide polymorphisms were selected from studies reported to be associated with CVDs or myocardial infarction (MI) [27]. All subjects from whom deoxyribonucleic acid (DNA) samples were available were expected to be genotyped for the rs1333048. The extraction of genomic DNA from blood samples was carried out with the use of the Gene ALL DNA kit (Type G Exgene; Genall; Korea) according to the manufacturer’s protocol. The concentration and purity of extracted DNA was measured using Nano Drop ND-1000 spectrophotometer. DNA was stored at − 20 °C until ready for use. The chromosome 9p21 rs1333048 SNP (major allele: A; minor allele: C) was genotyped by polymerase chain reaction-restricted length polymorphism (PCR–RFLP) technique. PCR was done using the following primers: forward 5′-ACCCGAAGTAGAGCTGCAAA-3′; reverse 5′-CACAAGTTGGAATATGAAGCAGA-3′. PCR reactions were performed in a final volume of 20 µl contains 2 µl extracted DNA, 0.5 µl primers, 10 µl distilled water and 7 µl Taq DNA Polymerase Master Mix (Ampliqon; Denmark) with the following conditions in a DNA thermocycler: The DNA templates were denatured at 94° C for 5 min (min); amplification consisted of 35 cycles at 94 °C, 63 °C and 72 °C (each step for 30 s), with a final extension at 72 °C for 7 min. Amplified DNA (10 µl) was digested with 2 µl of DRI restriction enzyme (Thermo fisher scientific; United states) at 37 °C overnight. All products visualized by electrophoresis in agarose gel. Fragments containing three possible genotypes were then distinguished: uncut homozygous CC (152 bp), cut heterozygous CA (84, 68 and 152 bp) and cut homozygous AA (84 and 68 bp). Ten percent of the samples were directly sequenced for confirmation the PCR–RFLP results.

### Statistical analyses

Normality distribution was tested by applying Kolmogorov–Smirnov’s test. Data on quantitative characteristics were reported as the mean ± SD and data on qualitative characteristics were expressed as a percentage. Qualitative variables were compared with analysis of variance (ANOVA) and independent *t* test to compare the quantitative variables. Moreover, age, physical activity, sex, BMI and energy intake-adjusted analyses were performed in general linear models (GLM). H-ORAC, L-ORAC, T-ORAC and TP intakes were stratified into two groups: low and high intake based on the median (defined as for H-ORAC, L-ORAC, T-ORAC, TP, respectively 31894.55 μmolTE/100 g, 38992.07 μmolTE/100 g, 70770.86 μmolTE/100 g, 2614.81 mgGAE/100 g). Mean values for the dietary variables were adjusted for total energy intake by using the residual method [27], then the study variables were compared among two groups using an independent T-test. Genotypes of markers were recoded based on risk allele: code 0 for CC, 1 for AC and 2 for AA genotype. In order to examine the interactions between rs1333048 genotype and TAC intakes on odds of MetS, the participants were grouped based on CDKN2B genotypes: group 1 with CC genotype (n = 76), group 2 with AC genotype (n = 127), and group 3 with AA genotype (n = 60). The binary logistic regression model was used to analyze potential interactions between rs1333048 genotype and TAC on odds of MetS. In this model, MetS criteria were entered as the dependent variable and rs1333048 genotypes and categorized intake of H-ORAC, L-ORAC, T-ORAC, and T based on median intakes were entered as covariates included in the crude model, and also adjusted for confunder variable also has been done at the next model. We find confounding factors with best fitted model and adjusted its effect of on exposure group, for interaction between rs 1333048 genotype and H-ORAC intakes on odds of MetS, adjusted for sex, age, physical activity and WC and for interaction between rs1333048 genotype and L-ORAC, T-ORAC and TP intakes on odds of MetS, adjusted for sex, age, physical activity and BMI. Confounding factors were determined from a best-fit model and the change in − 2 log likelihood ratio test. The level of significance was set at a probability of < 0.05 for all tests. Statistical analysis was performed using SPSS version 22.0 (SPSS, Chicago, IL, USA).

## Results

### Study population characteristics

This comparative cross-sectional study was conducted on 263 participants (52.5% female). The means (± SD) of age, height, BMI, and weight of individuals were 35.08 ± 8.78 years, 168.23 ± 9.43 cm, 25.93 ± 4.89 kg/m^2^, and 73.51 ± 15.66 kg, respectively (Table 1). The frequencies of A and C alleles of rs1333048 were 52.85% and 47.15% respectively. The overall prevalence of rs1333048 genotypes was 22.6%, 47.9% and 28.7% for AA, AC and CC respectively (Table 2). The distribution frequencies of mentioned SNP in this study followed Hardy–Weinberg equilibrium (P > 0.05). It was found that 12.5% of participants had MetS, and the data demonstrated that MetS across AA, AC, CC genotypes were respectively 13.3%, 11%, and 13% (P = 0.86).

**Table 1:** Study population characteristics

	Min	Max	Mean	SD^a^
Demography				
Age (year)	18	55	35.08	8.78
Weight (kg)	36.50	142.00	73.51	15.66
Height (cm)	148.00	193.50	168.23	9.43
Body composition				
BMI (kg/m^2^)	18.55	46.22	25.93	4.89
Fat percentage %	2.40	48.20	25.59	9.37
FFM (kg)	29.93	104.37	54.28	9
Fat mass (kg)	1.31	51.48	19.08	8.72
VFR	1	17	5.52	3.40
BMR	1050.00	2676.00	1603.34	324.27
Waist (cm)	58.00	130.00	88.80	12.50
Hip (cm)	68.00	144.00	102.62	9.57
Blood parameters				
FBS (mmol/l)	73.00	292.00	94.10	18.51
TG (mmol/l)	32.00	726.00	126.04	96.01
T-Chol (mmo/l)	109.00	433.00	184.58	40.33
HDL-C (mg/dl)	20.00	84.00	48.78	11.68
LDL-C (mg/dl)	44.00	282.00	101.28	27.22
hs-CRP (mg/l)	0.10	20.00	2.33	3.34
HOMA-IR	0.31	10.54	2.92	1.56
Blood pressure				
Systolic (mmHg)	17.30	11.94	1.28	17.30
Diastolic (mmHg)	10.90	7.73	0.91	10.90

FBS, fast blood sugar; TG, triglyceride; T-Chol, Total cholesterol; HDL-C, High density lipoprotein cholesterol; LDL-C, low density lipoprotein cholesterol; hs-CRP, high sensitivity C-reactive protein; HOMA-IR, Homeostatic model assessment-Insulin resistance; BMI, body mass index; FFM, free fat mass ;VFR, visceral fat rate; BMR, basal metabolic rate; Hip, Hip circumference, waist, waist circumference

^a^Standard deviation

**Table 2:** rs1333048 genotypes and allelic variants of study population

	Alleles frequency		Genotypes frequency		
	A	C	AA	AC	CC
rs1333048 genotypes	47.15%	52.85%	(n = 60) 22.6%	(n = 127) 47.9%	(n = 76) 28.7%

### Association between biochemical parameters, body composition, anthropometric measurements and rs1333048 genotypes

A total of 263 Iranian men and women were categorized based on rs1333048 genotypes and divided into three groups: CC genotype (n = 76), AC genotype (n = 127) and AA genotype (n = 60) (Table 3). Means of WC, hip, BMI, weight, FM, fat percentage and visceral fat rate (VFR) were higher in participants carrying the A allele, compared with individuals in the CC genotype, but that there was no statistically significant difference across three groups, even after adjustment for BMI, age, sex and physical activity. Also, no significant difference was observed regarding TG, T-chol, HDL-C, LDL-C, hs-CRP, systolic and diastolic blood pressure across groups, even after adjustment for confounding factors.

**Table 3:** Characteristics of study population according to rs1333048 genotypes

rs1333048 genotypes	CC (n = 76) Mean ± SD	AC (n = 127) Mean ± SD	AA (n = 60) Mean ± SD	P value	P value*
Demography					
Age (years)	34.47 ± 8.77	35.76 ± 8.92	34.35 ± 8.66	0.47	0.72**
Height (cm)	169.61 ± 9.44	167.75 ± 10.05	167.66 ± 7.60	0.34	0.42^a^
Weight (kg)	73.28 ± 14.55	72.93 ± 15.07	74.87 ± 18.20	0.73	0.29^2^
Body composition					
BMI (kg/m^2^)	25.51 ± 4.82	25.86 ± 4.69	26.51 ± 5.44	0.50	0.22^a^
Fat percentage %	24.00 ± 9.60	25.97 ± 9.74	26.68 ± 8.58	0.23	0.81^a^
Fat mass (kg)	18.05 ± 8.94	19.07 ± 8.65	20.34 ± 9.01	0.35	0.83^a^
FFM (kg)	55.71 ± 11.63	53.29 ± 11.46	54.33 ± 13.51	0.42	0.80^a^
VFR	5.30 ± 3.42	5.6460 ± 3.37247	5.55 ± 3.60	0.80	0.68^a^
BMR	1651.57 ± 340.66	1578.78 ± 317.83	1586.95 ± 310.22	0.31	0.33
Waist (cm)	88.16 ± 12.21	88.55 ± 12.32	89.82 ± 13.37	0.74	0.90^a^
Hip (cm)	102.43 ± 8.47	102.50 ± 9.70	103.08 ± 10.75	0.91	0.06^a^
Blood parameters					
FBS (mmol/l)	94.34 ± 17.07	95.16 ± 22.60	92.03 ± 7.57	0.55	0.88
TG (mmol/l)	121.25 ± 102.81	131.28 ± 104.70	119.01 ± 63.99	0.64	0.96
T-chol (mmol/l)	180.33 ± 37.89	189.43 ± 43.79	179.80 ± 35.43	0.17	0.94
HDL-C (mg/dl)	48.26 ± 12.51	49.26 ± 10.78	48.81 ± 12.52	0.84	0.88
LDL-C (mg/dl)	98.89 ± 24.00	104.10 ± 30.58	98.33 ± 23.44	0.27	0.90
hs-CRP (mg/l)	2.48 ± 3.96	1.97 ± 2.49	2.87 ± 4.01	0.20	0.76
HOMA-IR	2.79 ± 1.41	2.78 ± 1.46	3.42 ± 1.96	0.06	0.21
Blood pressure					
Systolic (mmHg)	11.80 ± 1.12	12.01 ± 1.33	12.00 ± 1.37	0.53	0.32
Diastolic (mmHg)	7.67 ± .93	7.78 ± .95	7.70 ± .80	0.72	0.97

FBS, fast blood sugar; TG, triglyceride; T-Chol, Total cholesterol; HDL-C, High density lipoprotein cholesterol; LDL, low density lipoprotein; hs-CRP, high sensitivity C-reactive protein; HOMA-IR, Homeostatic model assessment-Insulin resistance; BMI, body mass index; FFM, free fat mass;VFR, visceral fat rate; BMR, basal metabolic rate; Hip, Hip circumference, waist, waist circumference; SD, standard deviation; GLM, General Linear Model

* After adjustment for age, sex, BMI and physical activity

** Put out the collinear variable from the GLM as confounders

^a^BMI considered as collinear and this variable adjusted for Age, Sex and PA

### Dietary intake

After adjustment for calorie intake across rs1333048 genotypes, there were significant differences in protein (P < 0.001), polyunsaturated fat (P = 0.02), zinc (P = 0.04), phosphorus (P = 0.02), magnesium (P = 0.01), selenium (P = 0.03), vitamin B3 (P = 0.05), L-ORAC (P = 0.02), T-ORAC (P = 0.02) consumption, and marginal significant differences observed in H-ORAC (P = 0.09). The results indicate that mean intake of H-ORAC, L-ORAC, T-ORAC and TP in the AA genotype was lower than in the CC genotype: 25,663.52, 43,613.40, 66,045.60, and 2640.56, respectively. Also, after adjustment for BMI, age, sex, and physical activity, significant differences were observed between genotypes and the intake of H-ORAC, L-ORAC, T-ORAC, protein, zinc, phosphorus, magnesium, and selenium (P < 0.05) (Table 4).

**Table 4:** Dietary intake of study population according to rs1333048 genotypes

rs1333048 genotypes	CC	AC	AA	P value	P value*
	(n=76 )	(n=127 )	(n=60 )		
	Mean ± SD	Mean ± SD	Mean ± SD		
Macronutrient					
Energy	2207.08 ± 677.06	2151.12 ± 549.72	2323.82 ± 614.59	0.20	0.42
Carbohydrate	324.64 ± 30.20	325.68 ± 23.03	319.51 ± 24.45	0.31	0.36
Protein	79.22 ± 12.31	82.03 ± 9.66	85.52 ± 11.90	*< 0.001*	*< 0.001*
Fat	70.41 ± 13.37	69.24 ± 10.27	71.07 ± 10.85	0.56	0.99
Carbohydrate					
Sugar	105.78 ± 43.67	107.81 ± 38.03	114.36 ± 40.77	0.45	0.38
Sucrose	25.78 ± 16.02	23.95 ± 15.12	22.59 ± 13.11	0.47	0.30
Glucose	14.15 ± 6.56	12.77 ± 5.33	13.94 ± 5.39	0.19	0.54
Fructose	17.16 ± 8.20	15.60 ± 6.47	16.96 ± 6.60	0.24	0.22
Fatty acids					
Saturated	24.33 ± 11.33	22.95 ± 9.06	25.09 ± 9.10	0.34	0.96
Poly Unsaturated	13.76 ± 3.35	12.72 ± 3.74	14.77 ± 5.51	*0.02*	0.49
Mono Unsaturated	20.01 ± 8.37	19.32 ± 6.49	21.74 ± 7.45	0.11	0.38
Trans	0.0001 ± .00	0.0001 ± .00	0.0001 ± .00	0.76	0.44
TAC					
H-ORAC (μmolTE/100)	28256.75 ± 8430.45	28608.50 ± 9150.77	25663.52 ± 7219.13	0.09	*< 0.001*
L-ORAC (μmolTE/100)	49841.18 ± 15994.96	50226.96 ± 16689.09	43613.40 ± 12833.07	*0.02*	*0.04*
T-ORAC (μmolTE/100)	74982.15 ± 23337.82	75856.27 ± 25110.43	66045.60 ± 19363.57	*0.02*	*0.05*
TP (mgGAE/100g)	2820.58 ± 657.73	2855.39 ± 663.66	2640.56 ± 520.50	0.10	0.11
Mineral					
Ca	994.04 ± 385.12	1010.97 ± 369.41	1088.15 ± 354.72	0.31	0.07
Iron	17.17 ± 5.2	17 ± 4.5	18 ± 5.333	0.10	0.12
Zinc	11.79 ± 3.77	12.27 ± 3.81	13.45 ± 4.09	*0.04*	*0.02*
P	1386.73 ± 445.23	1461.26 ± 405.29	1609.19 ± 497.20	*0.02*	*0.02*
Mg	363.36 ± 126.93	381.63 ± 126.44	429.80 ± 141.61	*0.01*	*0.01*
Copper	1.67 ± 0.52	1.65 ± 0.46	1.86 ± 0.54	0.20	0.07
Se	122.31 ± 41.20	130.58 ± 42.46	143.27 ± 53.70	*0.03*	*0.01*
Vitamin					
E	9.71 ± 4.27	9.70 ± 3.09	10.71 ± 4.17	0.20	0.29
A	492.25 ± 275.36	447.43 ± 214.48	525.50 ± 255.01	0.11	0.77
C	98.94 ± 62.42	87.20 ± 55.52	100.51 ± 51.07	0.21	0.74
D	1.70 ± 1.25	1.69 ± 1.19	1.91 ± 1.29	0.51	0.48
B1	2.14 ± 0.68	2.14 ± 5.74	2.27 ± 0.65	0.41	0.33
B2	1.90 ± 0.64	1.92 ± 0.61	2.12 ± 0.63	0.09	0.13
B3	23.26 ± 6.99	22.91 ± 6.15	22.43 ± 7.37	*0.05*	0.08
B6	1.70 ± 0.53	1.71 ± 0.52	1.87 ± 0.54	0.12	0.14
B12	3.83 ± 1.67	3.98 ± 1.68	4.1 ± 1.31	0.46	0.26

Italic values indicate significance of p value (p < 0.05)

TAC, total antioxidant capacity; H-ORAC, hydrophilic oxygen radical absorbance capacity; L-ORAC, lyophilic oxygen radical absorbance capacity; T-ORAC, total -oxygen radical absorbance capacity; TP, total phenolic; μmolTE, μmol of Trolox equivalents ; mgGAE, mg gallic acid equivalents

* After adjustment for calories intake

### Association between biochemical parameters, body composition, anthropometric measurements and TAC

The results of the comparison indicated that means of WC, TG, and T-chol were reduced from the low intake to high intake dietary TAC, but there was no statistically significant difference, as shown in Tables 5 and 6.

**Table 5:** Characteristics of study population based on median intake H-ORAC and L-ORAC

Intake	H-ORAC				L-ORAC			
	Low Mean ± SD	High Mean ± SD	P value	P value*	Low Mean ± SD	High Mean ± SD	P value	P value*
Demography								
Age (years)	34.46 ± 8.55	35.40 ± 9.00	0.40	0.29	35.38 ± 8.76	34.48 ± 8.90	0.41	0.83
Height (cm)	168.31 ± 9.2	168.37 ± 9.75	0.96	0.82	168.83 ± 9.58	167.84 ± 9.43	0.40	0.10
Weight (kg)	73.03 ± 15.9	73.78 ± 15.67	0.70	0.79	73.50 ± 16.14	73.31 ± 15.43	0.92	0.63
Body composition								
BMI (kg/m^2^)	25.74 ± 5.03	25.96 ± 4.69	0.72	0.86	25.73 ± 5.01	25.97 ± 4.71	0.70	0.96
Fat percentage %	25.04 ± 9.61	25.89 ± 9.00	0.47	0.25	25.38 ± 8.99	25.56 ± 8.99	0.87	0.50
Fat mass	18.28 ± 8.4	20.01 ± 9.07	0.13	0.14	16.57 ± 8.24	21.74 ± 8.59	< *0.001*	< *0.001*
FFM	60.11 ± 11.76	47.48 ± 8.2	< *0.001*	< *0.001*	62.83 ± 9.80	44.74 ± 5.03	< *0.001*	< *0.001*
VFR	5.31 ± 3.53	5.59 ± 3.20	0.51	0.77	5.39 ± 3.53	5.52 ± 3.20	0.77	0.68
BMR	1602.46 ± 320.63	1603.89 ± 333.41	0.97	0.66	1605.92 ± 334.46	1600 ± 319.61	0.89	0.07
Waist (cm)	88.55 ± 13.07	88.53 ± 11.95	0.98	0.29	89.02 ± 13.11	88.06 ± 11.84	0.55	*0.03*
Hip (cm)	102.15 ± 9.77	102.70 ± 9.26	0.65	0.90	102.80 ± 9.74333	102.05 ± 9.27	0.53	0.10
Blood parameters								
FBS (mmol/L)	93.29 ± 20.6	94.75 ± 16.72	0.53	0.57	93.43 ± 20.61	94.62 ± 16.75	0.61	0.49
TG (mmol/L)	130.8 ± 108	119.3 ± 9.92	0.31	0.27	131.13 ± 108.82	118.94 ± 69.19	0.28	0.24
T-chol (mmol/L)	184.1 ± 40.5	183.89 ± 37.6	0.95	0.59	186.13 ± 41.34	181.92 ± 36.57	0.39	0.33
HDL-C (mg/dl)	48.3 ± 11.89	49.05 ± 11.53	0.62	0.45	48.62 ± 12.18	48.77 ± 11.22	0.92	0.78
LDL-C (mg/dl)	100.3 ± 26.7	101.51 ± 26.2	0.71	0.88	101.81 ± 27.64	100.01 ± 25.30	0.58	0.47
hs-CRP (mg/l)	2.22 ± 3.33	2.38 ± 3.36	0.69	0.41	2.35 ± 3.39	2.25 ± 3.30	0.80	0.95
HOMA-IR	2.94 ± 1.67	2.90 ± 1.50	0.87	0.84	2.82 ± 1.53	3.01 ± 1.63	0.44	0.40
Blood pressure								
Systolic (mmHg)	11.82 ± 1.33	12.0 ± 1.25	0.19	0.11	11.88 ± 1.32	11.99 ± 1.27	0.52	0.33
Diastolic (mmHg)	7.70 ± .98	7.75 ± .84	0.68	0.67	7.731 ± .93	7.72 ± 0.90	0.96	0.93

FBS, fast blood sugar; TG, triglyceride; T-Chol, Total cholesterol; HDL-C, High density lipoprotein cholesterol; LDL-C, low density lipoprotein cholesterol; hs-CRP, high sensitivity C-reactive protein; HOMA-IR, Homeostatic model assessment-Insulin resistance; BMI, body mass index; FFM, free fat mass ;VFR, visceral fat rate; BMR, basal metabolic rate; Hip, Hip circumference, waist, waist circumference; *standard deviation; TAC, total antioxidant capacity; H-ORAC, hydrophilic oxygen radical absorbance capacity; L-ORAC, lyophilic oxygen radical absorbance capacity; Total-ORAC, total -oxygen radical absorbance capacity; TP, total phenolic, GLM, general linear models

* After adjustment for calories intake. H-ORAC, L-ORAC, were stratified into two groups low and high intake based on median (defined as or cut point for H-ORAC, L-ORAC, respectively 31894.55 μmolTE/100g ,38992.07 μmolTE/100g)

**Table 6:** Characteristics of study population based on median intake T-ORAC and TP

Intake	T-ORAC				TP			
	Low Mean ± SD	High Mean ± SD	P value	P value*	Low Mean ± SD	High Mean ± SD	P value	P value*
Demography								
Age (years)	35.22 ± 8.774	34.65 ± 8.909	0.60	0.90	34.90 ± 8.55	34.97 ± 9.13	0.94	0.94
Height (cm)	169.14 ± 9.347	167.53 ± 9.6	0.17	0.14	168.95 ± 9.64	167.72 ± 9.35	0.30	0.30
Weight (kg)	73.82 ± 16.15	72.99 ± 15.41	0.67	0.63	74.05 ± 15.67	72.76 ± 15.92	0.51	0.51
Body composition								
BMI (kg/m^2^)	25.76 ± 5.04	25.95 ± 4.67	0.75	0.99	25.90 ± 4.85	25.79 ± 4.88	0.86	0.86
Fat percentage %	25.10 ± 9.76	25.84 ± 8.84	0.53	0.61	24.66 ± 9.50	26.28 ± 9.06	0.17	0.30
Fat mass	16.92 ± 8.27	21.38 ± 8.76	< *0.001*	< *0.001*	18.40 ± 8.34	19.92 ± 9.21	0.18	0.17
FFM	62.11 ± 10.45	45.47 ± 6.13	< *0.001*	< *0.001*	59.46 ± 12.34	47.93 ± 8.09	< *0.001*	< *0.001*
VFR	5.47 ± 3.59	5.43 ± 3.14	0.92	0.82	5.54 ± 3.51	5.37 ± 3.23	0.69	0.95
BMR	1617.07 ± 328.94	1589.30 ± 324.72	0.50	0.12	1628.19 ± 323.57	1578.18 ± 328.75	0.23	0.71
Waist (cm)	89.06 ± 13.20	88.02 ± 11.76	0.51	*0.05*	89.12 ± 12.60	87.97 ± 12.39	0.47	0.47
Hip (cm)	102.73 ± 9.74	102.13 ± 9.28	0.62	0.15	102.34 ± 9.39	102.52 ± 9.64	0.87	0.87
Blood parameters								
FBS (mmol/L)	93.40 ± 20.58	94.66 ± 16.79	0.59	0.41	93.44 ± 20.51	94.61 ± 16.92	0.61	0.61
TG (mmol/L)	132.25 ± 108.91	117.81 ± 68.83	0.20	0.26	135.23 ± 110.41	114.96 ± 66.07	0.07	0.07
T-chol (mmol/L)	185.71 ± 41.15	182.34 ± 36.83	0.49	0.43	185.97 ± 39.59	182.11 ± 38.50	0.43	0.43
HDL-C (mg/dl)	48.47 ± 12.34	48.92 ± 11.04	0.76	0.89	48.40 ± 11.77	48.99 ± 11.65	0.68	0.68
LDL-C (mg/dl)	101.48 ± 27.44	100.34 ± 25.53	0.73	0.64	101.48 ± 26.59	100.35 ± 26.42	0.73	0.73
hs-CRP (mg/l)	2.36 ± 3.39	2.24 ± 3.30	0.76	0.98	2.20 ± 3.18	2.40 ± 3.5	0.62	0.62
HOMA-IR	2.98 ± 1.65	2.85 ± 1.51	0.62	0.63	2.83 ± 1.44	3.00 ± 1.70	0.49	0.41
Blood pressure								
Systolic (mmHg)	11.86 ± 1.32	12.00 ± 1.27	0.41	0.16	11.86 ± 1.34	12.00 ± 1.24	0.40	0.08
Diastolic (mmHg)	7.71 ± .94	7.73 ± .88	0.85	0.67	7.71 ± .94	7.73 ± 0.88	0.86	0.57

FBS, fast blood sugar; TG, triglyceride; T-Chol, Total cholesterol; HDL-C, High density lipoprotein cholesterol; LDL-C, low density lipoprotein cholesterol; hs-CRP, high sensitivity C-reactive protein; HOMA-IR, Homeostatic model assessment-Insulin resistance; BMI, body mass index; FFM, free fat mass ;VFR, visceral fat rate; BMR, basal metabolic rate; Hip, Hip circumference, waist, waist circumference; *standard deviation; TAC, total antioxidant capacity; T-ORAC, total-oxygen radical absorbance capacity; TP, total phenolic, GLM, general linear models

* After adjustment for calories intake. T-ORAC and TP intake were stratified into two groups low and high intake based on median (defined as or cut point for Total-ORAC, TP, respectively; 70770.86 μmolTE/100g, 2614.81 mgGAE/100g)

### Genes by dietary interactions on MetS

The binary logistic regression model analysis was used to examine the interactions between rs1333048 genotypes and H-ORAC, L-ORAC, T-ORAC and TP intake on odds of MetS.

In the crude models, there was no significant interaction between rs1333048 genotypes and TAC on the odds of MetS. After adjusting for age, sex, BMI and physical activity, a significant interaction was observed between rs1333048 genotypes and high L-ORAC intake on reduce odds of MetS (OR = 0.24, 95% CI = 0.06–0.94, P for interaction = 0.04). Moreover, there was no significant interaction between rs1333048 genotypes and T-ORAC and TP intake on the odds of MetS, OR = 0.28, 95% CI = 0.07–1.10, P for interaction = 0.07 and OR = 0.58, 95% CI = 0.16–2.07, P for interaction = 0.40) respiratory. Also, After adjusting for age, sex, WC and physical activity, a significant interaction was observed between rs1333048 genotypes and high H-ORAC intake on reduce odds of MetS (OR = 0.26, 95% CI = 0.06–0.99, P for interaction = 0.04).

**Fig. 1:**
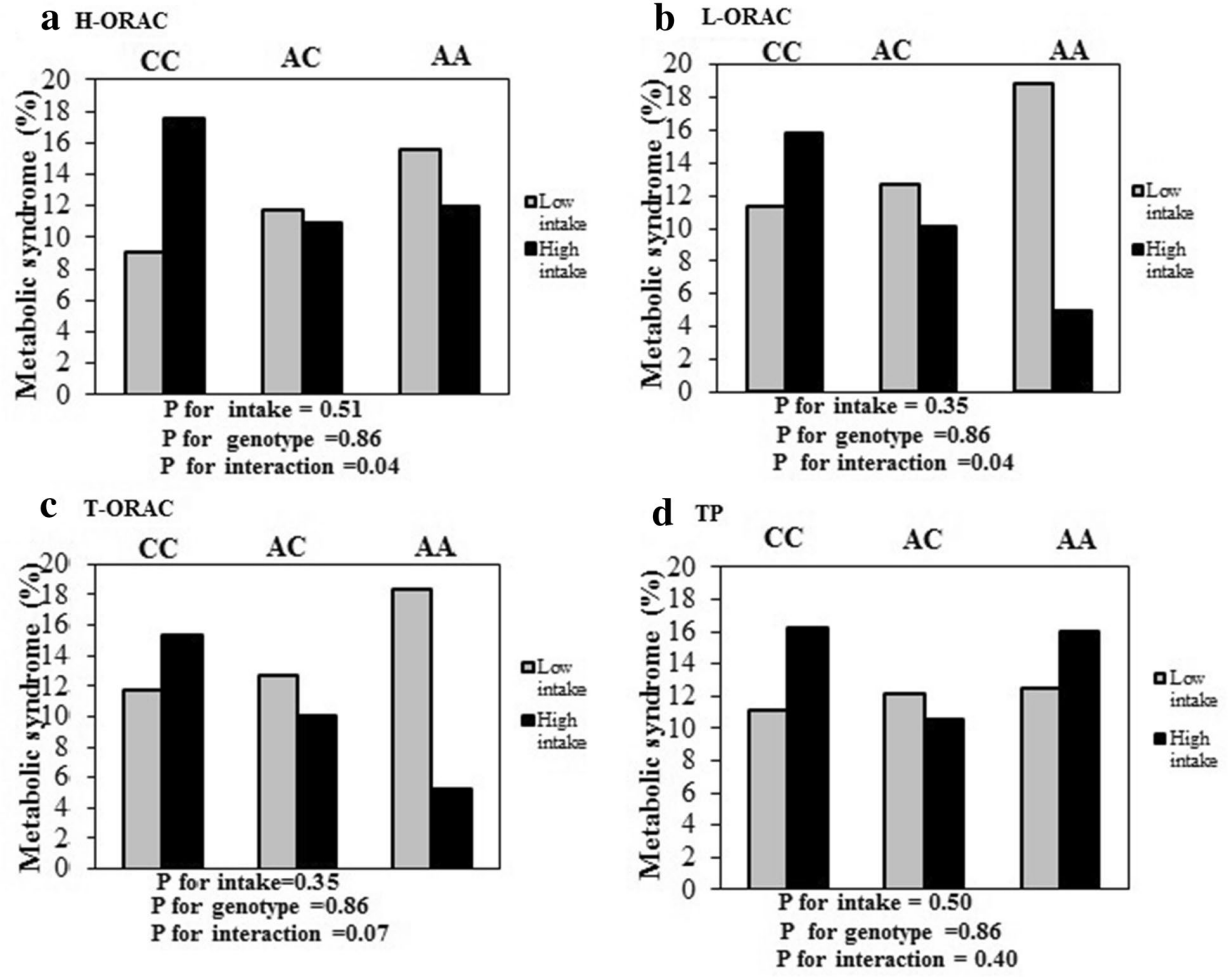
Percentage of MetS across rs1333048 genotypes base on low and high dietary TAC. Percentage of MetS across AA, AC and CC genotypes base on low and high dietary TAC. **a** Percentage of MetS across AA, AC and CC genotypes base on low and high H-ORAC intake. Percentage of MetS in low intake across AA, AC and CC genotypes were respiratory 15.6%, 11.7% and 9.1%. Percentage of MetS in high intake across AA, AC and CC genotypes were respiratory 12.0%, 10.9% and 17.5%. **b** Percentage of MetS across AA, AC and CC genotypes base on low and high L-ORAC intake. Percentage of MetS in low intake across AA, AC and CC genotypes were respiratory 18.9%, 12.7% and 11.4%. Percentage of MetS in high intake across AA, AC and CC genotypes were respiratory 5%, 10.1% and 15.8%. **c** Percentage of MetS across AA, AC and CC genotypes base on low and high T-ORAC intake. Percentage of MetS in low intake across AA, AC and CC genotypes were respiratory 18.4%, 12.7% and 11.8%. Percentage of MetS in high intake across AA, AC and CC genotype were respiratory 5.3%, 10.1% and 15.4%. **d** Percentage of MetS across AA, AC and CC genotypes base on low and high TP intake. Percentage of MetS in low intake across AA, AC and CC genotypes were respiratory 12.5%, 12.1%11.1%. Percentage of MetS in high intake across AA, AC and CC genotypes were respiratory 16.0%, 10.6% and 16.2%

Overall, in low L-ORAC intake, percentage of MetS across AA, AC and CC genotypes were 18.9%, 12.7%, and 11.4%, respectively. Whereas, in high L-ORAC intake, were 5%, 10.1%, and 15.8% respectively. Also, in high H-ORAC intake, percentage of MetS, across AA, AC and CC genotypes was 12%, 10.9%, and 17.5% respectively (Fig. 1).

In the general linear model (GLM) analysis, the associations of rs1333048 genotypes and H-ORAC, L-ORAC, T-ORAC and TP were tested on MetS components including: TG, HDL-C, LDL-C, T-chol, WC, and BP but there was no significant difference even after controlling for BMI, sex, age and physical activity.

## Discussion

The main finding was that rs1333048 polymorphism on chromosome 9p21 may be associated with a higher odds of MetS, and that high H-ORAC and L-ORAC intake can modify this association and reduce the odds of MetS.

Interestingly, an inverse and strong association was found between high dietary L-ORAC and H-ORAC intake and the odds of MetS across AA genotype. Generally, high dietary L-ORAC, H-ORAC and T-ORAC intake modified the association of the rs1333048 genotypes with the odds of MetS in the AA genotype. When stratified by rs1333048 genotypes, the high dietary L-ORAC and H-ORAC intake was only associated with a lower risk of MetS in individuals with risk alleles.

In the present study it was found that the AA genotype had mean higher fat mass and fat perecentage, although there was no statistical significance, and also that the prevalence of MetS in the AA genotype was higher than in the CC genotype. Teeuw et al. reported the frequency of rs1333048 genotypes was AA 35.7%, AC 46.6% and CC 17.9% [28]. Overall, frequency of the genotypes can vary in different populations. Several studies have shown the important role of the CDKN2A/B gene via the modulation of several pathways involved in adipocytes, pancreatic beta cells and less stable arterial plaque phenotypes [29]. Horswell et al. showed that the knockdown of CDKN2B expression in a mouse adipocyte cell line was associated with an increased level of adipogenesis [30], highlighting the importance of CDKN2B as determinant of adipogenesis.

The findings of the current study indicate that, overall, high TAC intake in the AA genotype reduces the following biochemical parameters: TG, FBS, LDL-C and hs-CRP (but without statistically significant differences). However, only high L-ORAC and T–ORAC intakes reduce WC in the AA genotype, in comparison with the CC and AC genotypes, with a statistically significant difference. In fact, an increased consumption of antioxidant-rich foods, such as fruits, vegetables, olive oil, nuts, and seafood resulted in an improvement in lipid profiles, with increased HDL-C and decreased LDL-C, in some intervention trial studies [31]. Some studies have shown the importance of oxidative stress in the physiopathology of obesity, and MetS has demonstrated that oxidative stress values increase with the number of components of MetS, and also that fat accumulation is closely correlated with markers of systemic oxidative stress [32, 33]. In fact, increased oxidative stress in accumulated fat leads to dysregulated adipocytokines production [34].

To the best of this team’s knowledge, this is the first study evaluating the interactions between genetic variants of the 9p21 locus with H-ORAC, L-ORAC, T-ORAC and TP. The current study indicates that the intakes of L-ORAC and T-ORAC in individuals with an AA genotype was lower in comparison with CC and AC genotypes, and there was a statistically significant difference. The results illustrated an interaction between rs1333048 A allele and high L-ORAC intake with a reduced odds of MetS. In fact, low L-ORAC intakes in subjects with A alleles increases the odds of MetS, but when L-ORAC and H-ORAC intake increases, it leads to a reduced odds of MetS in the AA genotypes, not seen in other genotypes. Also, T-ORAC intakes in the AA genotype reduces the odss of MetS, but with only marginal significant differences, while TP intake has no effect on the odds of MetS across all genotypes. The transcription factors in lipophilic binding in the CDKN2B gene promoters are PKNOX1, SOX5, ZSCAN4, and HDGF, which regulate cell cycles and coding proteins [35]. Thus, it was hypothesized that the mechanism of this interaction between the AA genotype and high L-ORAC intake with a reduced risk of MetS might be via transcription factor lipophilic, which can bind lipophilic antioxidants, including vitamin E and carotenoids. One regulatory mechanism of CDKN2A/B expression is through epigenetic mechanisms directed by ANRIL [36]. If epigenetic mechanisms contribute to the association between 9p21 and CVD, the current results suggest that they could potentially be influenced by nutritional or environmental factors and through gene-environment interactions. Milagro et al. reported possible associations between genetic variations at this locus with obesity as important risk factors for CVDs, as well as its association with environmental factors such as dietary intake [35]. Similarly, Qi et al. studied the interactions between genetic backgrounds and dietary patterns in diabetes subjects. They found that a western dietary pattern might elevate diabetes risk, particularly among subjects with mutations in this locus [36].

The favorable effects of these antioxidant-rich foods on the improvement of lipid profiles, glucose homeostasis, insulin resistance, adiposity and obesity have been investigated in pre-clinical and some clinical studies [37]. In addition, dietary antioxidants also affect other aspects of obesity-related metabolic pathways, including the inhibition of intestinal fat abortion, the promotion of catabolism in adipose tissue, the inhibition of proliferation, differentiation, and angiogenesis in pre-adipocytes, and the induction of apoptosis in mature adipocytes [38]. Some other dietary antioxidants could prevent adiposity by regulating brown adipose tissue metabolism and increasing thermogenesis, decreasing adiponectin and leptin gene expression in adipocytes [39].

Strength of this study is that it is the first study to evaluate the interaction between rs1333048 genotypes and TAC on the odds of MetS in subjects, and that it was a community-based study. The main limitation of the present study was the relatively small number of subjects and the low prevalence of MetS. Also, the cross-sectional design of the study, in which it was not possible to determine the mechanism of the relationship between L-ORAC/H-LORAC and the rs1333048 genotype could be considered as a limitation.

## Conclusion

The findings of this study suggest that high L-ORAC and H-LORAC intake can reduce the odds of MetS in the AA genotype. The present evidence indicates that this could be a novel link between TAC and rs1333048 genotypes. However, the mechanism of interaction between L-ORAC/H-ORAC and AA genotypes is not clearly understood. This study provides further evidence to recommend antioxidant-rich foods as a useful tool in health promotion and disease prevention.

## Abbreviations

ANOVA: analysis of variance
ATPIII: Adult Treatment Panel III
BIA: Bioelectrical Impedance Analyzer
BMI: body mass index
CHDs: chronic heart diseases
CVDs: cardiovascular diseases
CDKN2B: cyclin-dependent kinase inhibitor 2 B
DNA: deoxyribonucleic acid
DXA: dual energy X-ray absorptiometry
FFQ: food frequency questionnaire
FCT: food composition table
GLM: general linear model
GOD/PAP: glucose oxidase phenol 4-aminoantipyrine peroxidase
GPOPAP: glycerol-3-phosphate oxidase phenol 4-aminoantipyrine peroxidase
GWAS: genome-wide association studies
H-ORAC: hydrophilic oxygen radical absorbance capacity
HDL: high-density lipoprotein Cholesterol
hs-CRP: hypersensitive C-reactive protein
FPG: fasting plasma Glucose
LDL: low-density lipoprotein
L-ORAC: lyophilic oxygen radical absorbance capacity
MetS: metabolic syndrome
MI: myocardial infarction
ORAC: oxygen radical absorbance capacity
PCR–RFLP: polymerase chain reactions–restriction fragment length polymorphism
ROS: reactive oxygen species
SNPs: single-nucleotide polymorphisms
TAC: total antioxidant capacity
T-ORAC: total oxygen radical absorbance capacity
TP: total phenolic
T2D: type 2 diabetes
TG: triglycerides
T-chol: total cholesterol
VFR: visceral fat rate
WC: waist circumference
